# Dr. Vincent G. Hamill

**Published:** 1914-05

**Authors:** 


**Dr. Vincent G. Hamill,** Buffalo, 1884, died recently of heart disease in New York City. He was the fourth physician in his family line.

